# Voltammetric detection of sumatriptan in the presence of naproxen using Fe_3_O_4_@ZIF-8 nanoparticles modified screen printed graphite electrode

**DOI:** 10.1038/s41598-021-98598-1

**Published:** 2021-12-15

**Authors:** Somayeh Tajik, Mahboobeh Shahsavari, Iran Sheikhshoaie, Fariba Garkani Nejad, Hadi Beitollahi

**Affiliations:** 1 Research Center of Tropical and Infectious Diseases, Kerman University of Medical Sciences, Kerman, Iran; 2 Department of Chemistry, Faculty of Science, Shahid Bahonar University of Kerman, 76175-133 Kerman, Iran; 3 Environment Department, Institute of Science and High Technology and Environmental Sciences, Graduate University of Advanced Technology, Kerman, Iran

**Keywords:** Medical research, Chemistry, Materials science, Nanoscience and technology

## Abstract

A novel electrochemical sensing platform was designed and prepared for the simultaneous detection of sumatriptan and naproxen by exploiting the prowess of the Fe_3_O_4_@ZIF-8 nanoparticles (NPs); as-synthesized Fe_3_O_4_@ZIF-8 NPs were characterized by energy-dispersive X-ray spectroscopy, fourier transform infrared spectroscopy, X-ray diffraction, field emission scanning electron microscopy (FESEM), transmission electron microscopy and thermal gravimetric analysis. The immobilized Fe_3_O_4_@ZIF-8 NPs on a screen printed graphite electrode (SPGE) was evaluated electrochemically via cyclic voltammetry, linear sweep voltammetry, and differential pulse voltammetry as well as chronoamprometery means; Fe_3_O_4_@ZIF-8/SPGE exhibited good sensing performance for sumatriptan in a range of 0.035–475.0 µM with detection limit of 0.012 µM. Also, Fe_3_O_4_@ZIF-8/SPGE exhibited good sensing performance for naproxen in a range of 0.1–700.0 µM with detection limit of 0.03 µM. The modified electrode showed two separate oxidative peaks at 620 mV for sumatriptan and at 830 mV for naproxen with a peak potential separation of 210 mV which was large enough to detect the two drugs simultaneously besides being stable in the long-run with considerable reproducibility. Real sample analyses were carried out to identify the function of fabricated electrode in sensing applications wherein trace amounts of sumatriptan and naproxen could be identified in these samples.

## Introduction

Migraine is defined as a persistent, recurring, and complicated neurovascular complication which has about three times higher prevalence in females compared to the males^1^ and characterized by throbbing or pulsating headache, typically unilateral, with mild to acute intensities. The attacks may have various durations ranging at a few hours to several days, with different frequencies of one episode every year to four episodes every month. There are also other symptoms in migraine including nausea, vomiting, photophobia, phonophobia, osmophobia, loss of appetite, fatigue, diarrhea, blurriness of vision, nasal congestion, pallor, sweating, scalp tenderness, neck stiffness, as well as aura^2^. Migraine imposes considerable social impacts, affecting the quality of life as well as work productivity^3^. Over the years, different acute treatments have been provided for this complication. Currently, migraine therapies are categorized as specific (ergot derivatives as well as triptans) or non-specific (analgesics along with non-steroidal anti-inflammation medications)^4^.

Sumatriptan as a synthetic medication belongs to the triptan class, deployed in humans to treat migraine and cluster headache attacks^5^. Sumatriptan is a specific and vascular serotonin (5-hydroxytryptamine; 5-HT) type 1-like receptors selective agonist, probably the 5-HT1D and 5-HT1B subtypes. The binding of sumatriptan and serotonin type-1D receptors leads to vasoconstriction of considerably dilated cranial blood vessels along with alleviation of migraine-associated pains^6^. Nevertheless, an overdose would cause toxicity together with diverse side effects such as paresthesia, warm/cold sensations, chest pains, exhaustion as well as vertigo^7^. Because of critical and physiological significance of sumatriptan, the detection of this medication in a variety of samples particularly biological ones is of paramount interest.

Despite the increasing application of the triptans, the non-steroidal anti-inflammatory drugs are still distributed at the highest frequency to treat acute migraines such as naproxen as effective nonspecific analgesic and anti-inflammatory medication, prescribed for diverse pains and inflammatory syndromes, namely migraines^8^. In this impediment, the drug’s analgesic effects relieve the headache on one hand, while the anti-inflammatory effects decrease the neurogenic inflammations in the trigeminal ganglion on the other hand. In general, involuntary naproxen intake as residues in foods brings about health risks for individuals, comprising allergies, serious gastrointestinal lesions, alterations in renal function as well as nephrotoxicity^9^. The vital contribution of naproxen in humans’ health emphasizes its determination in biological samples.

Numerous analytical methods for determining these compounds (sumatriptan and naproxen) have been reported, including spectrophotometry^10,11^, capillary electrophoresis^12,13^, high performance liquid chromatography (HPLC)^14,15^, HPLC with fluorescence detection^16^, liquid chromatography–mass spectroscopy (LC–MS)^17^, high performances thin layer chromatography (HPTLC)^18^, spectrofluorimetry^19^, chemiluminescence^20^, and electrochemical detection using voltammetric techniques^21–23^.

Given the complimentary mode of sumatriptan and naproxen action, their integrated application would present more desirable clinical advantages compared to their single application in acute migraine therapies. Hence, the simultaneous detection of these medications in biological fluids and pharmaceutical formulations is very important^24^.

Among the various methods of detection drugs, electrochemical methods are noteworthy candidates for analyzing drugs, as they are versatile, portable, and capable of system miniaturization system, with no compromise in terms of sensitivity and selectivity^25^. The electrochemical methods employ low-cost instruments in comparison to the instrumentation needed in chromatographic or spectrophotometric methods, while the low detection limit and lower reagent usage are additional beneficial attributes^26,27^.

Currently, screen printed electrodes (SPEs) are garnering attention as they have noticeably challenged the traditional three electrode cell systems. Such devices can be accessed through printing on a plastic or ceramic support a series of inks which contain the components of the working, reference as well as auxiliary electrode along with the necessary connectors. The appliance of SPEs offers as an uncomplicated, disposable, nontoxic and inexpensive option compared with other solid electrodes, and employed to quantify a diverse set of substances^28^. Their versatile design and the option to employ a broad range of printing ink compositions, along with the convenient improvement of their surface are regarded as salient features of such devices. Moreover, it is easy to adapt them to flow and automated systems, and their easy connection to mobile instruments expedite in place analyses^29^.

The use of unmodified SPEs for the electrochemical detection generally results in low sensitivity and selectivity due to the drawbacks of unmodified electrodes including loss of surface passivation, high background noise, heterogeneous surface, non-repeatability of surface conduct, overpotential requirement, slow kinetics of electrochemical reactions of some compounds onto the surface of the electrode, and may incur large interferences from other existing electroactive species in real samples limiting the applicability of unmodified electrodes for the analytical usages.

Chemically modified electrodes (CMEs) based electrochemical sensors reflect a future trend in analytical chemistry^30^. In a variety of contexts, the direct electrochemical detection on a “bare” or unmodified electrode takes place just at higher cathodic or anodic potentials. However, a considerable passivation effect of the electrode, brought on by species formed over the electrochemical process, would poison the electrode thus necessitating the modification of the electrode’s surface to solve or diminish such challenges. Furthermore, the modification of the surface increases the electrode kinetics while enhancing the detection sensitivity as well as selectivity^31,32^.

To date, a wide range of electrode modifiers such as metal and metal oxide NPs, conducting polymers, carbon nanotubes, graphene, ordered mesoporous carbon, and so on have been considered as modifiers in electrochemical sensors. Recently, metal organic frameworks (MOFs), or as they are often termed coordination networks or polymers, including metal ions or clusters of metals as nodes and organic units connected by coordinate bonds, are getting significant attention in applied sciences. MOFs are widely considered desirable candidates to modify the electrode surface because of their unprecedented features, such as considerable surface area, adjustable pore sizes, flexible structure, chemical integrity and convenient functionalization as well as the design^33,34^. Zeolitic imidazolate frameworks (ZIFs), known as a nitrogen containing subclass of MOFs, are novel porous inorganic–organic hybrid crystalline materials consisting of metal ions or metal clusters bridged tetrahedrally by imidazolate-type linkers^35^. ZIFs are good candidates in the field of electrochemical sensing due to their good dispersion and high adsorbability to small molecules^36^.

Magnetite (Fe_3_O_4_) as one of the most commonly used magnetic nanomaterials have attracted researchers from different fields including catalysts, absorbents, sensors, wastewater treatments, magnetic resonance imaging (MRI), as well as drug delivery, due to their multifunctional properties such as small size, biocompatibility, catalytic activity, superparamagnetism, low toxicity, and easy preparation. Fe_3_O_4_ NPs have the capability to enhance electrode conductivity and facilitate electron transfer^37^. Also, the Fe_3_O_4_@ZIF-8 exhibits synergistic effect and thus improve the performance for electrochemical sensor construction.

Based on above considerations, herein, a sensitive and simple voltammetric sensor is described constructed as Fe_3_O_4_@ZIF-8/SPGE to simultaneously detect sumatriptan and naproxen. It exhibited excellent electrochemical performance with lower detection limit, extended linear range, good stability and reproducibility. Moreover, the applicability of this sensor toward sumatriptan and naproxen detection in real samples was evaluated. Furthermore, to the best of our knowledge, the simultaneous detection of sumatriptan and naproxen via electrochemical means has been unprecedented yet.

## Experiments

### Apparatus and chemical substances

The use of Autolab potentiostat/galvanostat (PGSTAT 302N, Eco Chemie, the Netherlands) helped to measure electrochemicals. The use of General Purpose Electrochemical System (GPES) software aimed at controlling the experimental conditions. Moreover, SPGE (DropSens; DRP-110: Spain) possessed 3 typical graphite counter, unmodified graphite working, and silver pseudo-reference electrodes. Metrohm 710 pH meter was utilized for pH measurements. EDS was performed using the MIRA3 instrument. Measurement of FT-IR spectra was carried out using a Shimadzu 8400 spectrometer. Examination of XRD patterns was accomplished with XRD device model X'Pert Pro made in the Netherlands. The samples’ morphology was observed through a FESEM-FEI Nanosem 450 field emission scanning electron microscope (FE-SEM) as well as a Philips EM208S 100 kV transmission electron microscope (TEM) at 120 kV, correspondingly. TGA was measured by using STA 503 instrument. The surface area along with the adsorption–desorption isotherm was determined on a BELSORP MINI II at 77 K with the use of liquid nitrogen as the coolant, while degassing of the samples at 473 k for 1. Sumatriptan, naproxen and other analytical grade reagents were acquired from Merck (Darmstadt, Germany). Orthophosphoric acid and the associated salts with a pH ranging higher than 2.0–9.0 have been utilized to prepare the buffer solutions.

### Preparation of Fe_3_O_4_ NPs

Fe_3_O_4_ nanoparticles were synthesized by a simple method. In a normal preparation method, 0.912 g FeCl_3_·6H_2_O and 1.9 g sodium acetate with 0.52 g trisodium citrate were dissolved in 40 ml ethylene glycol under magnetic stirring. After that, the mixed solution was transferred into a Teflon-lined stainless-steel autoclave and placed in an oven to be heated to 200 °C for 10 h. After the autoclave was cooled to room temperature, the Fe_3_O_4_ nanoparticles were separated by a magnet from solution, subsequently washing with deionized water and methanol several times. The resulting product was dried at 60 °C for 14 h.

### Preparation of Fe_3_O_4_@ZIF-8

In a typical procedure, at first 0.050 g of Fe_3_O_4_ spherical NPs were mixed with 0.821 g methyl imidazole (MeIM) in 25 mL methanol via sonication. Then, 25 mL of methanol solution containing 0.3689 g of Zn(NO_3_)_2_·6H_2_O was quickly added and sonication for 5 min until it became homogeneous. The suspension was kept under reflex (60 °C) for 2 h. Finally, using an external magnet, the product was separated and washed with deionized water and dried at 60 °C for 5 h^39^.

### Electrode preparation

Modification of the screen printed working electrode was accomplished using Fe_3_O_4_@ZIF-8 NPs through a simple drop casting technique. To prepare the Fe_3_O_4_@ZIF-8 NPs stock solution in 1 mL of aqueous solution, the Fe_3_O_4_@ZIF-8 NPs (1 mg) was dispersed for 30 min via ultrasonication. After that, a 5 µl Fe_3_O_4_@ZIF-48 NPs suspension was dropped onto the surface of screen printed working electrode. Then, the evaporation of the solvent was allowed at the ambient temperature.

### Real sample preparation

Five sumatriptan tablets (labeled 50 mg per tablet, Poursina Company, Iran) were ground. Next, the preparation of tablet solution was achieved by dissolving 50 mg of the powder in 25 mL water using ultrasonication. Later, varying quantities of the diluted solution were delivered into a 25 mL volumetric flask, diluted to the mark with PBS (pH 7.0) and the analysis were performed using modified electrode.

Five naproxen tablets (labeled 500 mg per tablet, Pharmashimy Company, Iran) were ground. Next, the preparation of tablet solution achieved by dissolving 500 mg of the powder in 25 mL water with ultrasonication. Later, the varying quantities of the diluted solution were delivered into a 25 mL volumetric flask, followed by dilution to the mark with PBS (pH 7.0) and analysis were performed using modified electrode.

## Result and discussions

### Characterizations

#### *EDX analyses of Fe*_*3*_*O*_*4*_*@ZIF-8 NPs*

**Figure 1:**
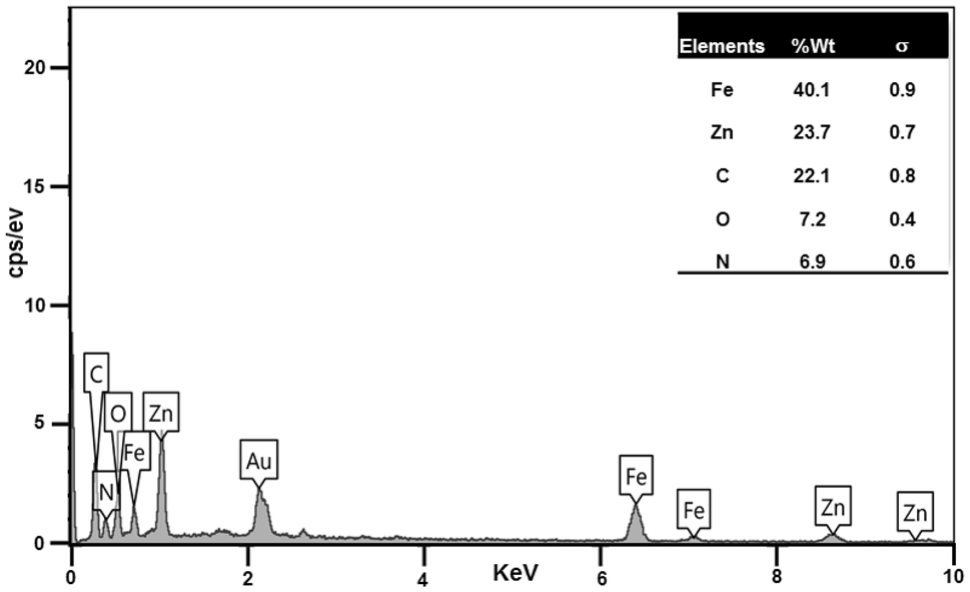
EDX spectrum of Fe_3_O_4_@ZIF-8 NPs.

The chemical compositions of Fe_3_O_4_@ZIF-8 NPs analysed by EDS that shown in Fig. 1. This pattern displayed that this NP is pure. It can be seen that the Fe, Zn, C, O and N exist in Fe_3_O_4_@ZIF-8 at the ratio of 40.1, 23.7, 22.1, 7.2 and 6.9 (%), respectively (Fig. 1).

#### FT-IR spectrum of Fe_3_O_4_@ZIF-8 NPs

**Figure 2:**
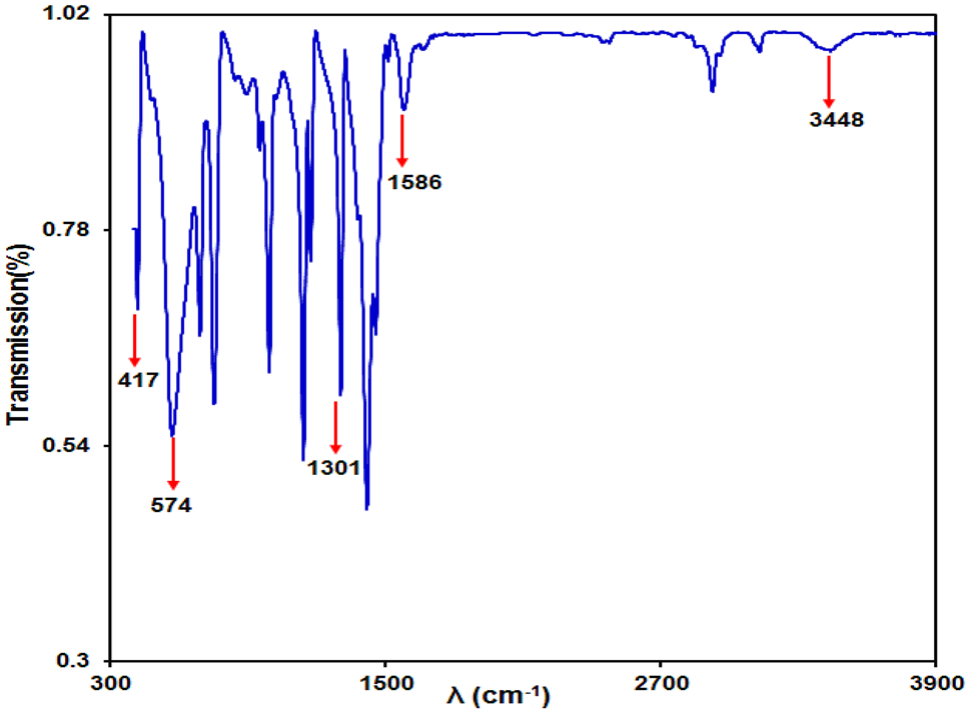
The Fe_3_O_4_@ZIF-8 NPs FT-IR spectrum.

The FT-IR spectra for the Fe_3_O_4_@ZIF-8 NPs are exhibited in Fig. 2. The bands at 1301 cm^−1^ and 1586 cm^−1^ correlate with carboxylate groups on the Fe_3_O_4_ NPs surface. In addition, the peak at 574 cm^−1^ may be associated with the Fe–O bonds. Additionally, the 417 cm^−1^ peak arises from the Zn–N stretch mode. Other bands in 1584 cm^−1^ and in the region of 690–1500 cm^−1^ are attributed to C=N stretch mode and imidazole ring, respectively. The peaks at 3101 and 2926 cm^−1^ are associated with the C–H stretching vibration in region aromatic as well as aliphatic bonds.

#### XRD patterns

**Figure 3:**
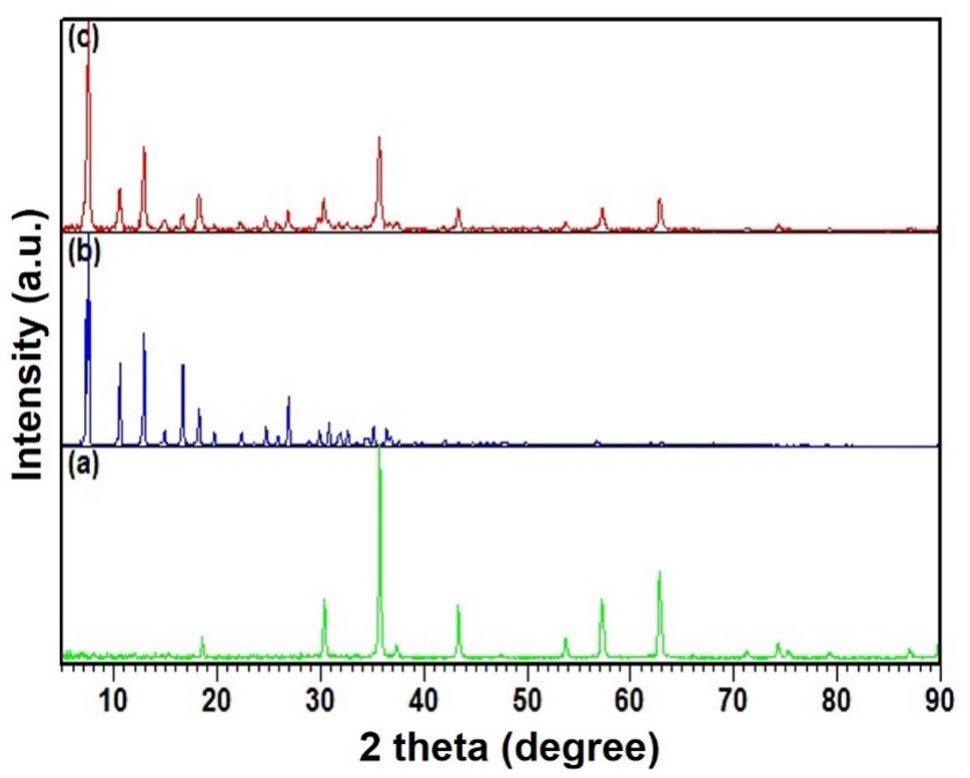
X-ray diffraction patterns of (**a**) Fe_3_O_4_ NPs, (**b**) ZIF-8, (**c**) Fe_3_O_4_@ZIF-8 NPs.

The diffraction peaks in Fe_3_O_4_@ZIF-8 XRD patterns confirmed the formation crystallite structure of magnetite NPs. Three dominant peaks at 7.4° (011), 10.3° (002), 12.9° (112) and 18.0° (222) of Fe_3_o_4_@ZIF-8 are well match to the JCPD Cod 00-062-1030 that is indicative of the retention of ZIF-8 in NPs; all particles exhibited pure crystals with average crystal size 17 nm. It is possible to correlate the diffraction peaks of the Fe_3_O_4_@ZIF-8 NPs at 2θ = 35.5°, 43.3°, 57.1° and 62.6°, with (311), (400), (511), (440) that show consistency with the Fe_3_O_4_ purity phase (JCPDS no.19-0629) (Fig. 3).

#### Morphology of Fe_3_O_4_@ZIF-8

**Figure 4:**
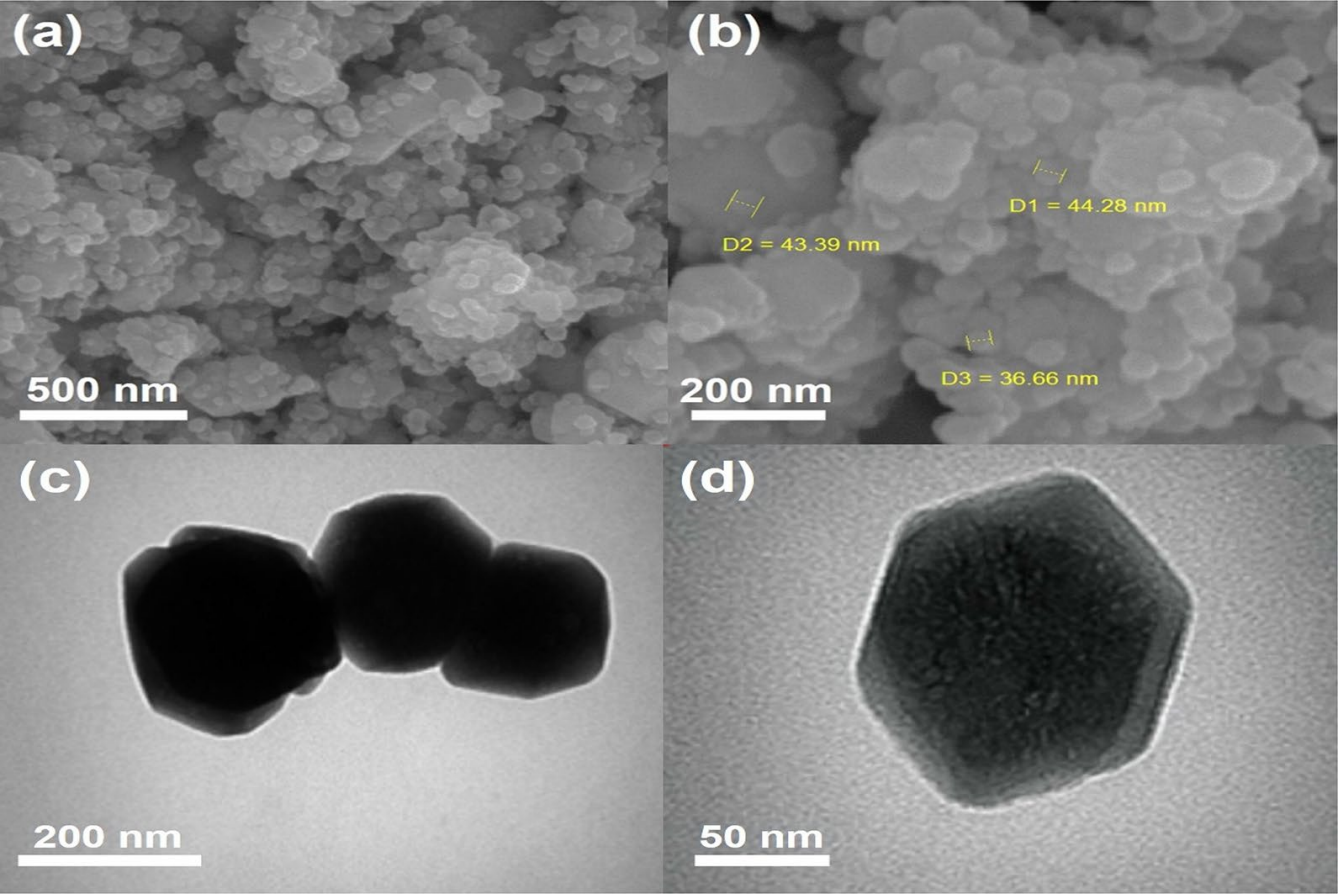
FESEM images of Fe_3_O_4_@ZIF-8 with average diameters of 36.66, 43.39 and 44.28 nm, at magnification of 500 and 200 nm (**a**,**b**). TEM images Fe_3_O_4_@ZIF-8 NPs at magnification of 200 and 50 nm (**c**,**d**).

The morphologies of Fe_3_O_4_@ZIF-8 are depicted in Fig. 4a,b. In details, the micrograph of NPs shows that the morphology has a hexagonal shape and these rough surfaces have average size 41.42 nm. As shown in Fig. 4c,d, the Fe_3_O_4_ crystals are microstructure and their size is less than 100 nm. According to these studies, ZIF-8 NPs have a positive charge because of the presence of self-stable imidazole ligands and also the presence of carboxylate groups in magnetic NPs have created a negative charge on the Fe_3_O_4_ surface; Fe_3_O_4_ NPs exhibit stability in MeIM solution. Therefore, when Zn(NO_3_)_2_ particles are added to a stable solution of MeIM and Fe_3_O_4_, a uniform core–shell structure with a magnetic core and a zeolite shell can be created.

#### TGA/DTA analysis

**Figure 5:**
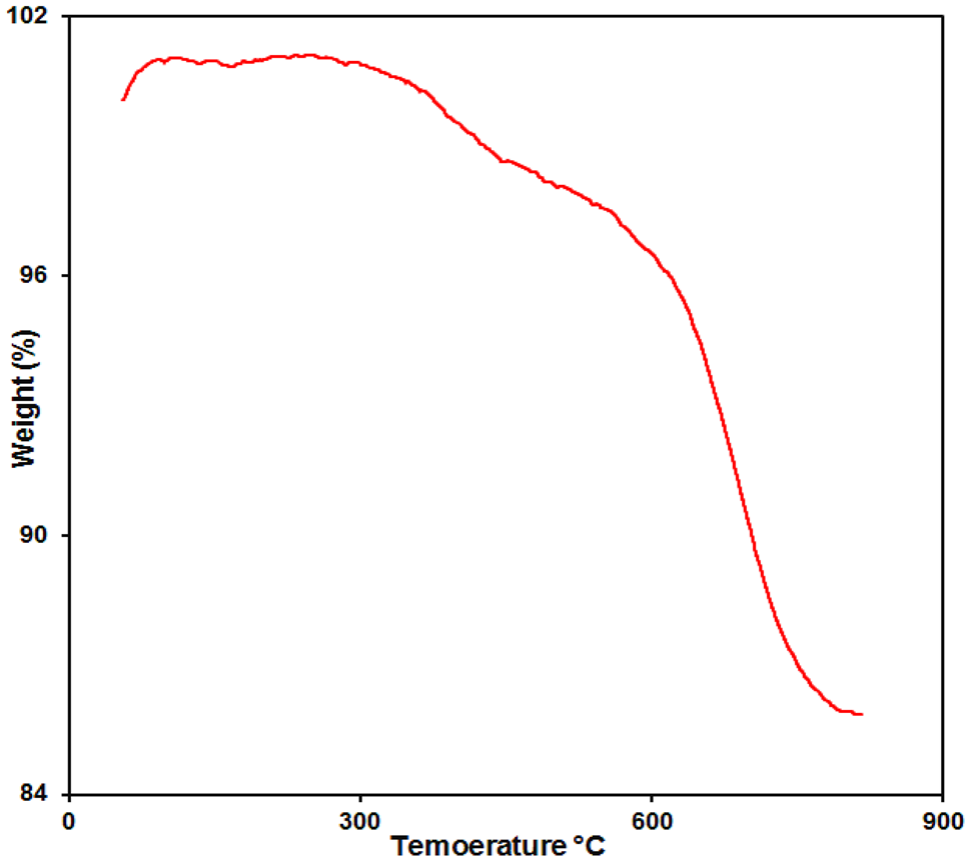
TGA under air of the Fe_3_O_4_@ZIF-8 NPs.

TGA curve for the Fe_3_O_4_@ZIF-8 are shown in Fig. 5. The first step was observed at 387 °C, that correspond to the removal of solvent molecules or guest molecules occluded within sample and is observed for only 2% weight loss up to 500 degrees. The second step, which has a steeper slope, related to the decomposition of the NPs, which occured at a temperature of 569 °C and lasts up to 800 °C. From above TGA data it was affirmed that the sample has extremely high thermal stability.

#### BET analysis

**Figure 6:**
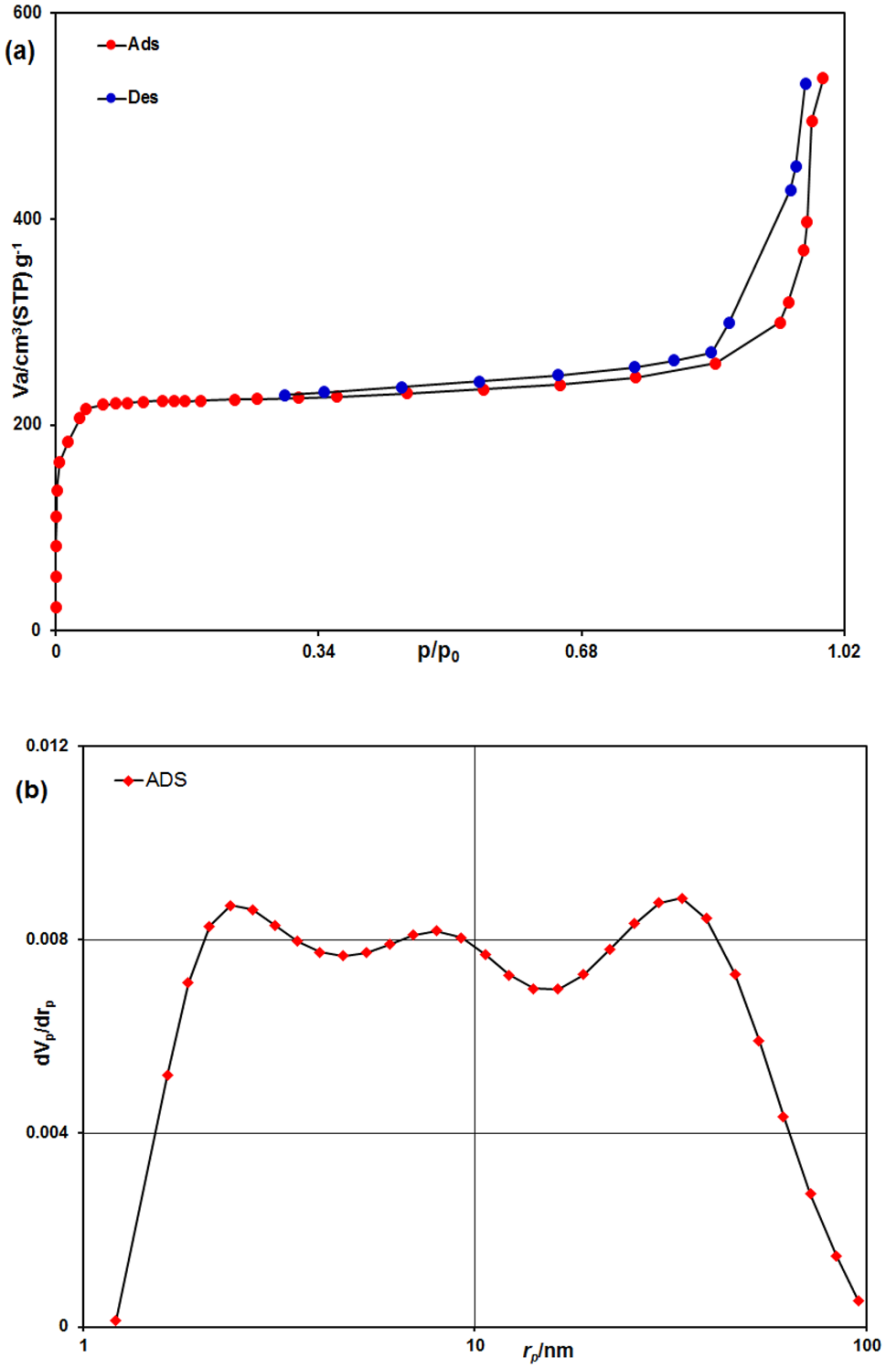
(**a**) Nitrogen gas adsorption–desorption isotherm of Fe_3_O_4_@ZIF-8 NPs. (**b**) BJH pore size distribution of Fe_3_O_4_@ZIF-8 NPs.

The Brunner-Emmet-Teller measurement (BET) technique facilitates the measurement of the material’s porosity. Thus, the Fe_3_O_4_@ZIF-8 porosity features and specific surface area (a_s,lang_) were assessed by performing BET. The porous NPs, based on IUPAC classification system, the adsorption isotherm can be categorized as type IV. Nitrogen (N_2_) absorption/desorption isotherms (77 K) with (BET) surface areas of 787.89 m^2^/g for Fe_3_O_4_@ZIF-8 is depicted in Fig. 6a. The BJH (Barrett-Joyner-Halenda) analyses indicate that the average diameter of the NPs is 3.79 nm, showing that porosity is present in the nanoscale (Fig. 6b).

### Electrochemical analysis

#### Electrochemical features of sumatriptan on the Fe_3_O_4_@ZIF-8/SPGE

To study electrochemical behaviours of sumatriptan, which was supposed to show dependence on pH, obtaining an optimum pH-value for the acceptable outcomes is important. Thus, the modified electrode was employed in conducting the experiments in 0.1 M phosphate buffer solution (PBS) in various pH-values between 2.0 and 9.0. Eventually, the most desirable results were considered for electrooxidation of sumatriptan at the pH of 7.0.

**Figure 7:**
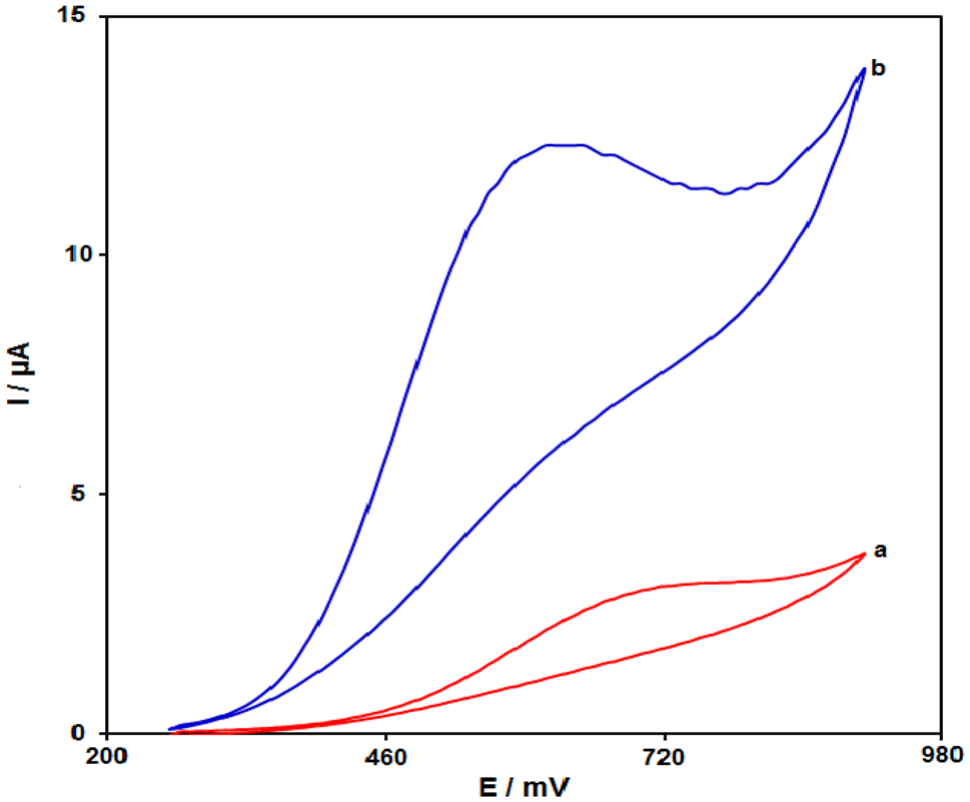
The CVs of (**a**) unmodified SPGE, (**b**) Fe_3_O_4_@ZIF-8/SPGE in 0.1 M PBS at pH of 7.0 when 100.0 µM sumatriptan is present at 50 mV s^−1^ scan rate.

Figure 7 depicts the cyclic voltammograms (CVs) for sumatriptan oxidation, at bare SPGE (a), and Fe_3_O_4_@ZIF-8/SPGE (b) in 0.1 M PBS (pH 7) solution including 100.0 µM sumatriptan at 50 mV s^−1^ scan rate. According to Fig. 7, a high overvoltage for electron transfer reaction of sumatriptan on the surface of unmodified SPGE was obtained. On the other hand, the high anodic peak current for sumatriptan was obtained using Fe_3_O_4_@ZIF-8/SPGE in comparison to unmodified SPGE. Hence, these results indicate high efficiency of Fe_3_O_4_@ZIF-8 NPs in electrocatalytic activities regarding the sumatriptan oxidation. The results of electrochemical responses of sumatriptan at unmodified SPGE and Fe_3_O_4_@ZIF-8/SPGE surfaces shown in Table 1.

**Table 1:** Comparison the response of unmodified SPGE and Fe_3_O_4_@ZIF-8/SPGE to 100.0 µM sumatriptan in PBS (pH 7.0); scan rate 50 mV s^−1^.

Electrode	Anodic peak potential (mV)	Anodic peak current (μA)
Fe_3_O_4_@ZIF-8/SPGE	760	12.3
SPGE	620	3.1

#### Scan rate effect

**Figure 8:**
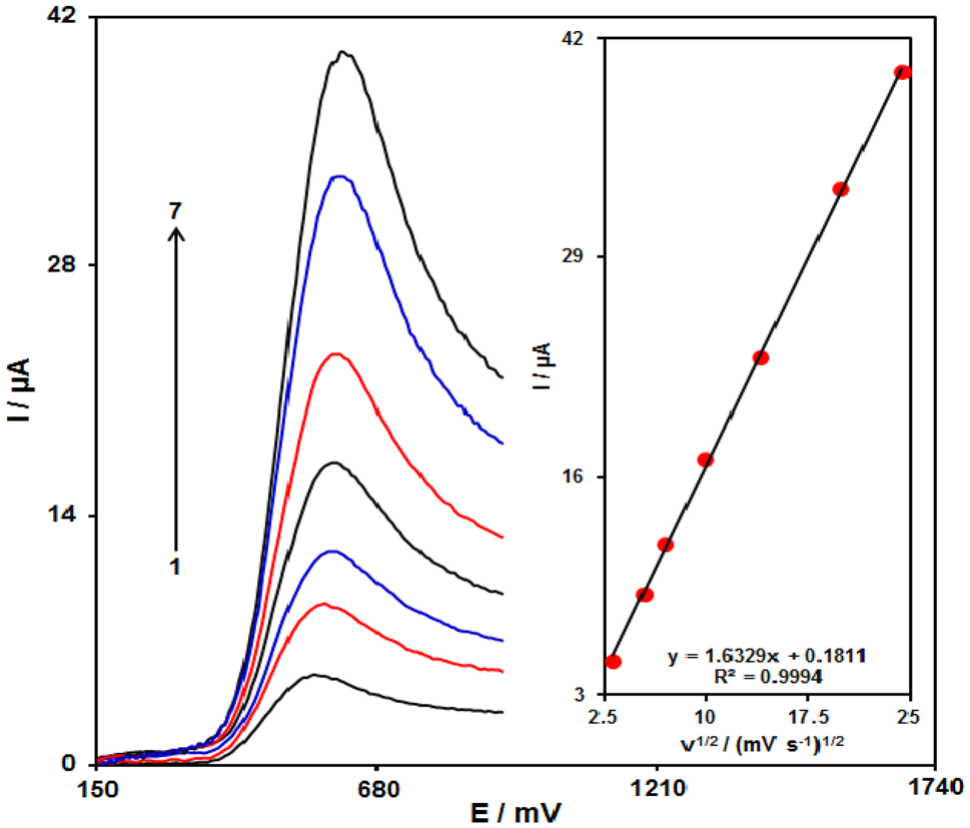
Linear sweep voltammograms (LSVs) of Fe_3_O_4_@ZIF-8/SPGE in 0.1 M PBS at pH of 7.0 including 100.0 µΜ sumatriptan at distinct scan rates; 1–7 respective to 10, 30, 50, 100, 200, 400, and 600 mV s^−1^. Inset; variations in the anodic peak currents versus ν^1/2^.

The association between peak current and scan rate would supply helpful information considering the underlying electrochemical mechanisms. Therefore, the scan rate effects on the peak current of 100.0 μM of sumatriptan were examined using LSV, at a range of 10–600 mVs^−1^ in PBS (0.1 M, pH 7), according to Fig. 8. The electrode response of sumatriptan was a diffusion-controlled procedure, as the oxidation peak current corresponded to the square root of the scan rate (Fig. 8 inset).

**Figure 9:**
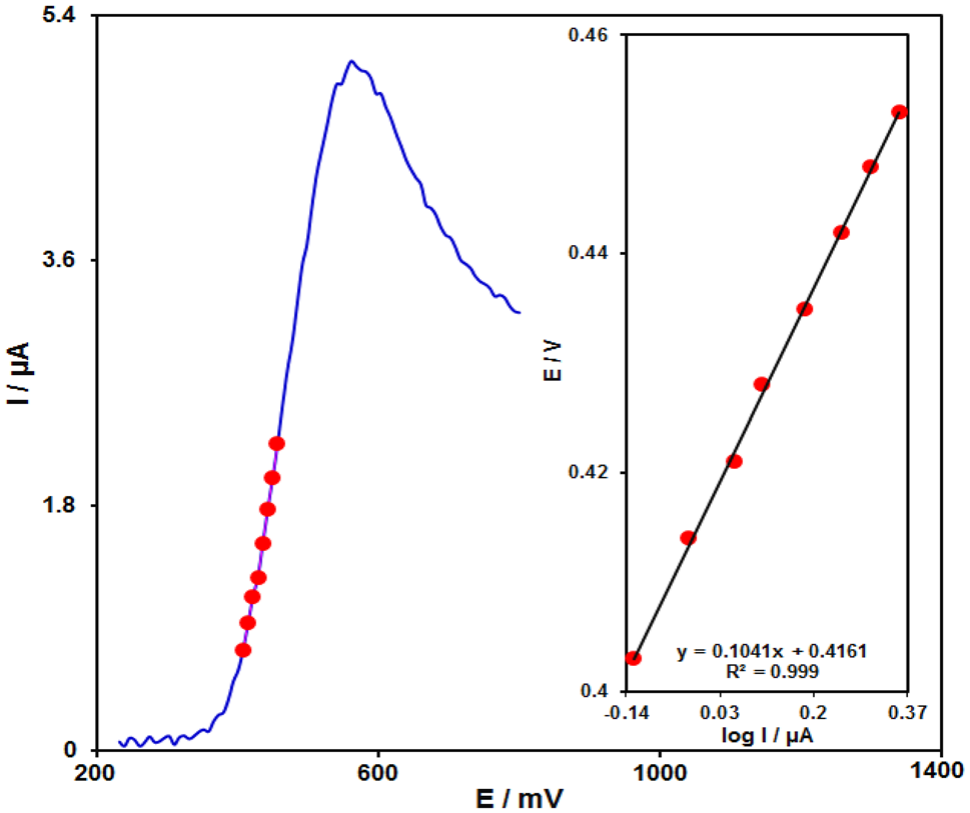
The LSV at 10 mV s^−1^ of the Fe_3_O_4_@ZIF-8/SPGE in 0.1 M PBS at pH of 7.0) with 100.0 µM sumatriptan. The points indicate the output used in Tafel plot. As can be observed, inset indicated Tafel plots obtained by the linear sweep voltammogram.

Figure 9, indicates a Tafel plot taken from data which were collected from the current rising part against the voltage curve recorded at 10 mV s^−1^ scan rate. It is notable that this part of voltammogram, called the Tafel region, was under the influence of the electron transfer kinetics of the substrate (sumatriptan) and Fe_3_O_4_@ZIF-8/SPGE. From the Tafel plot slope, the number of electrons engaged in the rate-identifying stage can be estimated. A 0.1041 V slope could be achieved for sumatriptan. Supposing an electron transfer in the rate-identifying stage, charge transfer coefficient (α) was estimated to be 0.43 for sumatriptan.

#### Chronoamperometric analysis

**Figure 10:**
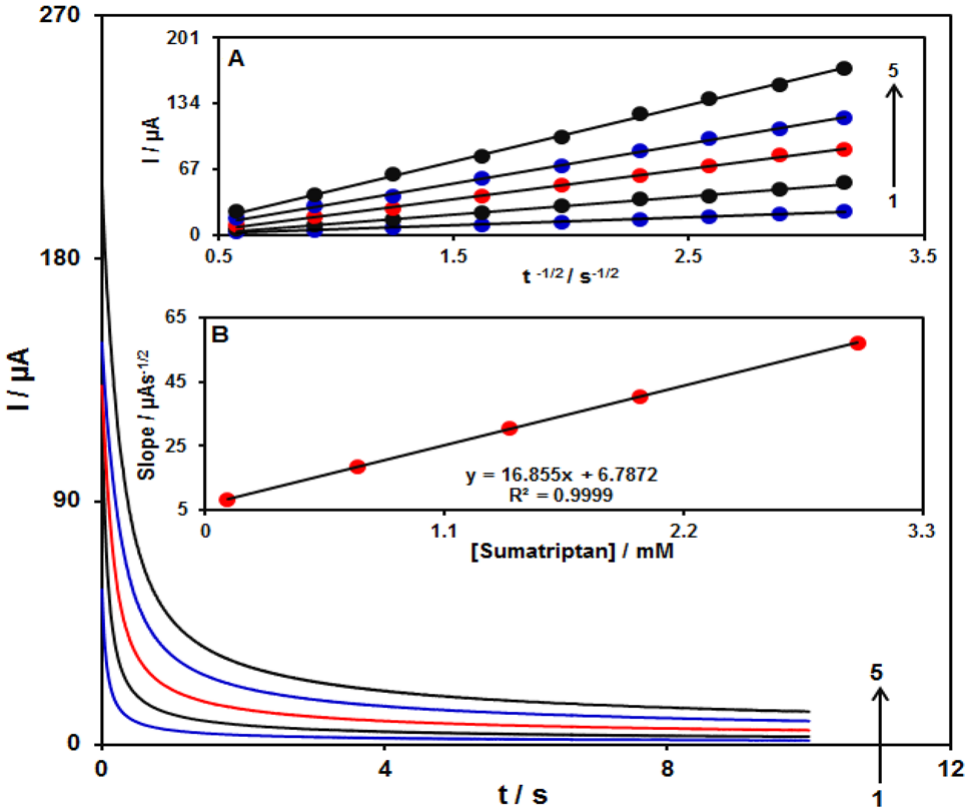
The chronoamperograms obtained at Fe_3_O_4_@ZIF-8/SPGE in 0.1 M PBS at pH of 7.0 for sumatriptan various concentrations. Accordingly, 1–5 relate to 0.1, 0.7, 1.4, 2.0 and 3.0 mM of sumatriptan. Inset A. the I plot versus t^−1/2^ observed using chronoamperograms 1–5. B. The straight-line slope plot versus sumatriptan concentration.

Chronoamperometric study was used for calculating the diffusion coefficient (D) of sumatriptan at the surface Fe_3_O_4_@ZIF-8/SPGE at an optimum condition. Figure 10 displays chronoamperometric outputs of the sumatriptan sample different concentrations in (PBS at pH of 7.0). In addition, Cottrell equation was recommended to perform electroactive moiety chronoamperometric analyses according to the mass transfer restricted conditions^40^:

$${\text{I}} = {\text{nFAD}}^{1/2} {\text{C}}_{{\text{b}}} \pi^{ - 1/2} {\text{t}}^{ - 1/2}$$

Figure 10A indicates the experimental findings regarding I versus t^−1/2^, which shows the most acceptable fit for the sumatriptan distinct concentrations. Then, the ultimate slopes relative to the straight lines in Fig. 10A could be depicted versus sumatriptan concentrations (Fig. 10B). Thus, D mean value equaled to 9.7 × 10^−5^ cm^2^/s regarding Cottrell equation and resultant slopes.

#### Calibration curve

**Figure 11:**
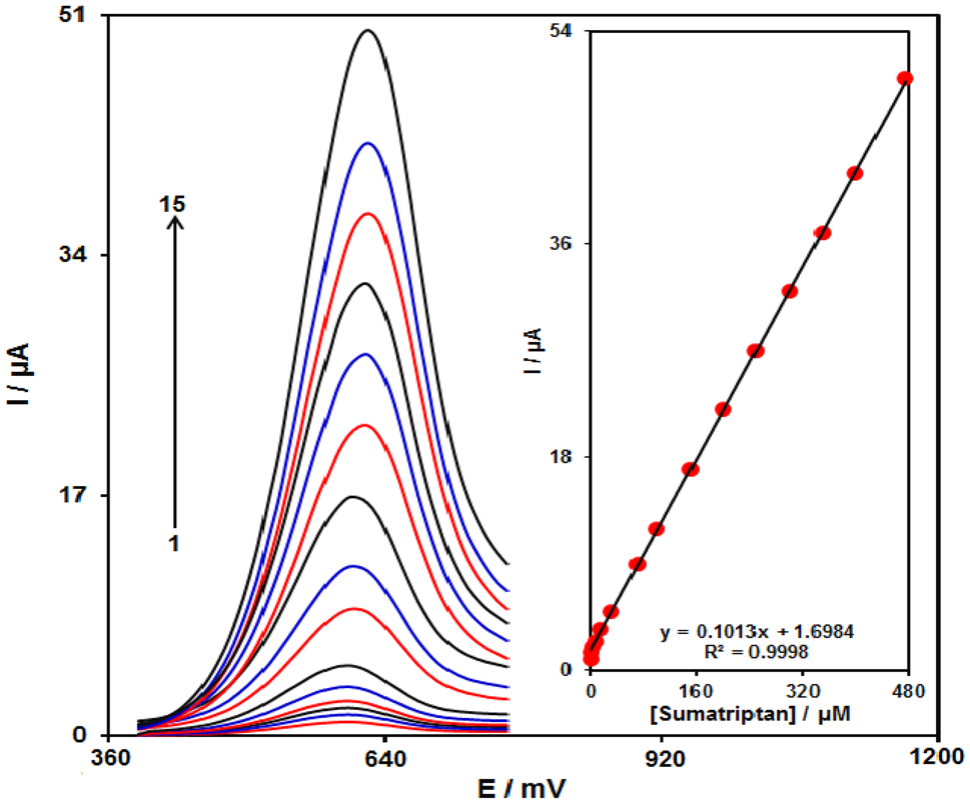
DPVs of Fe_3_O_4_@ZIF-8/SPGE in 0.1 M PBS (pH 7.0) including sumatriptan varying concentrations. 1–15 indicate 0.035, 0.2, 2.5, 7.5, 15.0, 30.0, 70.0, 100.0, 150.0, 200.0, 250.0, 300.0, 350.0, 400.0 as well as 475.0 μM of sumatriptan, respectively. The inset indicates the peak current plot as a function of the sumatriptan concentrations ranging from 0.035 to 475.0 μM.

The DPV method explored the association of the peak current with sumatriptan different concentrations. As shown in Fig. 11, the differential pulse voltammograms (DPVs) of Fe_3_O_4_@ZIF-8/SPGE in the presence of different concentration of sumatriptan was recorded in the concentration range varying from 0.035 to 475.0 μM (Step potential = 0.01 V and pulse amplitude = 0.025 V). The sensor based on Fe_3_O_4_@ZIF-8/SPGE exhibited excellent performance for sumatriptan detection, giving a low limit of detection (0.012 μM), limit of quantification (0.035 μM) and high sensitivity (0.1013 μA μM^−1^).

In the case of naproxen the DPVs of Fe_3_O_4_@ZIF-8/SPGE in the presence of different concentration of naproxen was recorded in the concentration range varying from 0.1 to 700.0 μM (Step potential = 0.01 V and pulse amplitude = 0.025 V). The sensor based on Fe_3_O_4_@ZIF-8/SPGE exhibited excellent performance for naproxen detection, giving a low limit of detection (0.03 μM), limit of quantification (0.1 μM) and high sensitivity (0.0733 μA μM^−1^).

#### Sumatriptan and naproxen simultaneous detection

**Figure 12:**
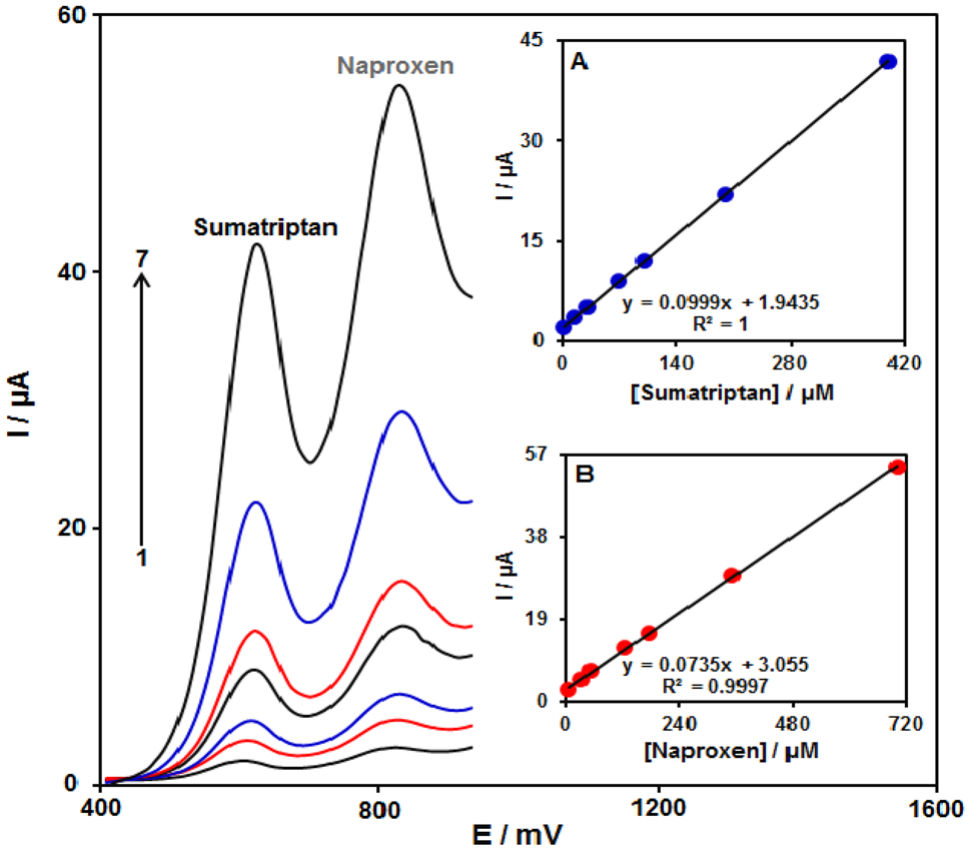
The DPV of the Fe_3_O_4_@ZIF-8/SPGE in 0.1 M PBS at pH of 7.0 using sumatriptan and naproxen varying concentrations. Accordingly, 1–7 relate to 2.5 + 5.0, 15.0 + 30.0, 30.0 + 50.0, 70.0 + 125.0, 100.0 + 175.0, 200.0 + 350.0, as well as 400.0 + 700.0 µM of sumatriptan and naproxen, respectively. Inset A. The Ip plot versus sumatriptan concentration. B. The Ip plot versus naproxen concentration.

The DPVs of sumatriptan and naproxen at Fe_3_O_4_@ZIF-8/SPGE in a solution, which had various concentrations of each, were recorded (Fig. 12) (Step potential = 0.01 V and pulse amplitude = 0.025 V). Two separated oxidation signals were apparent having potentials of ~ 620 mV (sumatriptan) and 830 mV (naproxen), which seemed adequate to simultaneously detect sumatriptan and naproxen. The current-concentration curves associated with sumatriptan and naproxen can be observed in Fig. 12A,B.

#### Stability and repeatability of Fe3O4@ZIF-8/SPGE

The stability of Fe_3_O_4_@ZIF-8/SPGE was evaluated using DPVs of the oxidation of 50.0 µM sumatriptan in first day and after two weeks (each measurement was done 5 times and the mean value was used). A 2.7% deviation was identified with compression of the first oxidation signal of sumatriptan following 2 weeks, indicating good stability of Fe_3_O_4_@ZIF-8/SPGE as a voltammetric sensor.

Examination of the Fe_3_O_4_@ZIF-8/SPGE antifouling features regarding sumatriptan oxidation and the corresponding products was carried out through DPV for the Fe_3_O_4_@ZIF-8/SPGE in first use and after successive fifteenth uses for 50.0 µM sumatriptan. The currents were reduced by lower than 2.4%, while the peak potential faced no alterations.

#### Real samples analysis

**Figure 13:**
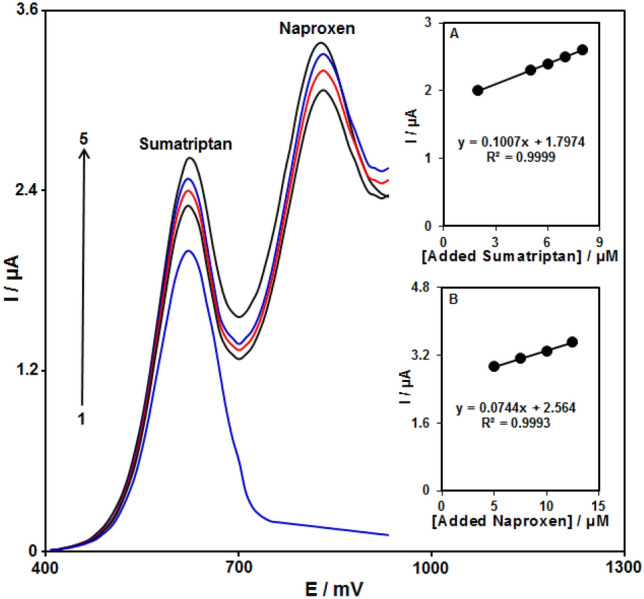
DPVs observed at Fe_3_O_4_@ZIF-8/SPGE surface in sumatriptan tablet sample of pH = 7.0 (0.1 M PBS) with distinct concentrations of sumatriptan as well as naproxen. As seen, the DPV numbers 1–5 has been relative to 2.0 + 0.0, 5.0 + 5.0, 6.0 + 7.5, 7.0 + 10.0 and 8.0 + 12.5 µM sumatriptan and naproxen.

Fe_3_O_4_@ZIF-8/SPGE performance as a new electrochemical sensor was used to analyze sumatriptan and naproxen in tablet samples. The results showed a sensitive sensor to analyze sumatriptan and naproxen in actual samples. Also, the data in Table 2 indicate that the results obtained by utilizing Fe_3_O_4_@ZIF-8/SPGE are in good agreement with those declared in the label of the preparations. Thus, the modified electrode can be efficiently used for individual or simultaneous determination of sumatriptan and naproxen in pharmaceutical preparations. The voltammograms of the sumatriptan tablet analysis are shown in Fig. 13.

**Table 2:** Comparison of the total values of sumatriptan and naproxen of some pharmaceutical preparations obtained using Fe_3_O_4_@ZIF-8/SPGE with declared values in the lable of the samples (n = 5).

Sample	Declared value	Obtained value	[Obtained concentration/ Declared value] × 100	RSD%
Sumatriptan Tablet (mg per tablet)	50	50.05	100.10 ± 0.02	2.4
Naproxen Tablet (mg per tablet)	500	500.37	100.07 ± 0.03	2.5

## Conclusions

In this work, a Fe_3_O_4_@ZIF-8 NPs has been utilized as a novel electrode modifier material for the detection of sumatriptan. Electrochemical sensing assays indicated the obvious catalytic abilities of Fe_3_O_4_@ZIF-8/SPGE in oxidizing the sumatriptan. An extensive linear range (0.035–475.0 µM), low detection limit (0.012 µM), high sensitivity (0.1013 μA μM^−1^), good stability as well as repeatability have been obtained through the development of sensor to analyze sumatriptan. Also, the simultaneous detection of sumatriptan and naproxen was also performed by means of the DPV method; peak separation of about 210 mV clearly allows the simultaneous detection of these drugs. Finally, sumatriptan and naproxen analyses in real samples validated the potential utility of this sensor and good recoveries (98–103.7%) were obtained from different spiked values.
